# Pediatric Burns: Biological and Tissue Engineered Skin Substitutes—A Systematic Review

**DOI:** 10.3390/jcm14227981

**Published:** 2025-11-11

**Authors:** Pietro Susini, Martina Certini, Gianluca Marcaccini, Ruggero Mazzotta, Roberto Cuomo, Giuseppe Nisi, Luca Grimaldi, Flavio Facchini

**Affiliations:** 1 Plastic Surgery Unit, Department of Medicine, Surgery and Neuroscience, University of Siena, 53100 Siena, Italy; 2 Meyer Children’s Hospital IRCCS, 50139 Florence, Italy; 3 Careggi University Hospital, 50134 Florence, Italy; 4 Department of Experimental and Clinical Medicine, University of Florence, 50134 Florence, Italy

**Keywords:** plastic surgery and pediatric burn, regenerative medicine and pediatric burn, allograft and pediatric burn, integra and pediatric burn, fish dermis and pediatric burn

## Abstract

**Background/Objectives**: Surgical debridement and early excision of burned areas followed by skin autograft is the gold standard of treatment for partial and full-thickness pediatric burns. However, skin autografting might be unfeasible or unlikely to succeed due limited availability of skin donor areas or inadequate conditions. In these circumstances, alternative treatment is required, and Skin Substitutes (SS) cold play a role. Recently, Biological Skin Substitutes (BSS) and Tissue Engineered Skin Substitutes (TESS) are emerging as alternative treatment options, but strong evidence is missing. This review investigates the current literature focusing on BSS and TESS, aiming to improve the medical and surgical management of pediatric patients. **Methods**: A systematic review was performed in accordance with the PRISMA 2020 guidelines and registered in the PROSPERO database (CRD42024627569). A comprehensive search was conducted in PubMed (MEDLINE) from 2000 to 2024 using Boolean logic and PICO-based inclusion criteria. Study quality was assessed using the Joanna Briggs Institute (JBI) critical appraisal checklists according to study design. **Results**: Twenty-nine articles and 2676 pediatric patients undergoing surgical reconstruction by BSS or TESS for burns were included. The methodological quality was generally moderate, with most studies being observational or case series. Several strategies were critically analyzed and possibly discussed. **Conclusions**: While BSS and TESS are safe and effective reconstructive options, the overall level of evidence remains low to moderate. A schematic classification of SS for pediatric burns is presented. Further prospective trials are needed to define standardized algorithms for pediatric burn reconstruction.

## 1. Introduction

The inherent curiosity, lack of experience, and compulsiveness of children lead to over 250,000 pediatric burns in the United States and over 2000 cases in Italy each year, accounting for approximately 22% of all hospital admissions for burns [1,2]. Thermal injuries such as scalds and flames are the main causes [3,4]. Recognized risk factors include improper adult supervision, child abuse, crowding, lack of education, and poor economic and social background [3,4]. When compared to adults, children present with thinner skin, immature kidneys, and a higher ratio of body surface area to body mass, ultimately resulting in increased risk of complications such as dehydration, hypothermia, dyselectrolytemia, and shock [2].

Currently, surgical debridement and early excision of burned areas, along with skin replacement with autograft, is the gold standard of treatment for partial-thickness and full-thickness pediatric burns [2,5]. Meshed grafts are typically required to obtain larger grafts, with a ratio up to 1:4–1:6 compared to the donor site [6]. The grafting procedure inherently requires a secondary wound at the harvesting site, representing an additional site for potential fluid loss, infection, and scars [7]. The Meek micrografting, based on small autograft islands regularly distributed or sprayed/glued over extended burns, has also been proposed to increase the donor site ratio from 1:6, up to 1:12 [8,9,10,11]. However, the Meek technique relies on a secondary intention to complete wound closure [12]. Consistently, extended burns of the face, neck, perineal, or hand should not be candidates for this procedure, considering the esthetic and functional sequelae [13,14]. In addition, although autografting of a third-degree burn is technically feasible, direct grafting of adipose tissue or muscle fascia causes skin retractions and an increased risk of pathological scarring, potentially leading to unsatisfactory outcomes. Overall, in some cases, skin autografting may be contraindicated or unlikely to succeed due to inadequate local or systemic conditions as well as lack of donor areas [2,5]. In these circumstances, Skin Substitutes (SS) could play a role.

Temporary and permanent SS of biological or tissue-engineered origin are available [15,16]. In major pediatric burns, these have been related to early wound coverage, fewer infections, pain reduction, lower metabolic stress, and shorter hospitalization [15,16]. Among these, allograft and xenograft are widely adopted [17,18,19,20]. However, there are limitations in terms of graft availability, risk of rejection, disease transfer, and cultural/ethical concerns [21].

Recently, technological advances have occurred in the field of burn wound care, and new biosynthetic SS, dermal regeneration models, and cultured/non-cultured autologous skin engineering products have been tested with promising results [22,23]. With the present paper, Biological Skin Substitutes (BSS) and Tissue Engineered Skin Substitutes (TESS) in pediatric burns are systematically reviewed, focusing on the latest technologies, aiming to improve the medical and surgical management of pediatric patients.

## 2. Materials and Methods

### 2.1. The Data Sources and Search Strategy

According to the PRISMA statement for Systematic Reviews [24,25], a comprehensive search of the literature was conducted on the PubMed (MEDLINE) library from January 2000 to October 2024 (Supplemental Digital Content 1). The Boolean search string was: (“Plastic surgery” OR “Regenerative medicine” OR “Allograft” OR “Skin equivalent” OR “Integra” OR “Fish dermis”) AND (“Pediatric burn”). An extensive list of terms to describe the target population based on the PICO acronym was formulated:

P (population)—Pediatric burns.

I (intervention)—Surgical reconstruction: BSS and TESS.

C (comparator)—Control group: absence of reconstruction, autograft, and medical treatment.

O (outcomes)—Assessment of any beneficial roles from BSS and TESS.

This systematic review was registered in the International Prospective Register of Systematic Reviews (PROSPERO), ID: CRD42024627569.

The search strategy was limited to PubMed to ensure inclusion of the peer-reviewed biomedical literature with robust indexing and clinical relevance. This methodological choice has been explicitly discussed in the Section 4.6.

### 2.2. Study Selection

The inclusion criteria were original studies (observational studies, randomized controlled trials, and case reports) reporting the use of BSS and TESS for the treatment of pediatric burns. Various aspects were focused on, including average healing time and perioperative complications. Since our focus was the use of SS, studies that specifically referred to autografting for pediatric burns were excluded. Studies were also excluded if they were animal studies, review articles or meta-analyses, books and documents, letters to the editor, or papers not written in English.

The literature search was performed by one independent reviewer (PS). Following title and abstract screening, we established whether publications met the selection criteria. Furthermore, when the title and abstract screening alone were unclear, the full text was reviewed and compared to the selection criteria. The bibliographical references were also screened. The included articles were then subjected to a full-text review and tested with the selection criteria. After study selection, data extraction, and critical appraisal, the collected data were brought to the attention of the senior author (FF) for final approval and possible dispute resolution. Accordingly, the selected papers were re-examined and finally included to present the information of this review.

### 2.3. Risk of Bias and Quality Assessment

In accordance with PRISMA recommendations, the methodological quality and risk of bias of included studies were assessed using the Joanna Briggs Institute (JBI) critical appraisal checklists, tailored to each study design (case report, case series, cohort, or RCT).

A summary of the quality assessment is presented in Supplementary Table S1, indicating study type, JBI score, and main sources of bias.

This step was added to enhance methodological transparency and to strengthen the systematic nature of the review.

### 2.4. Data Extraction and Analysis

Data were extracted on patient demographics (age and gender), Total Body Surface Area (TBSA) of burns, and surgical procedures. Outcomes of interest included surgical strategies and technical details. Moreover, we evaluated the perioperative complications, and we calculated the average healing time, aiming to assess the feasibility of the procedures. All quantitative data (healing time, complications, and TBSA) were standardized using uniform units (days and %TBSA). When multiple studies reported the same outcome, mean values were calculated as weighted averages based on the number of patients per study. Studies lacking sufficient outcome data or using non-comparable measures were excluded from the pooled averages to avoid statistical bias.

Separate outcomes were obtained for articles on BSS and TESS. The role of autografting, the treatment of the long-term complications, and survival rates were not endpoints of the study and were not considered in this article.

Overall, this review focuses on innovative surgical strategies for pediatric burns and evaluates the specific indications and potential benefits of each technique, with the aim of improving the medical and surgical management of these patients.

## 3. Results

**Figure 1:**
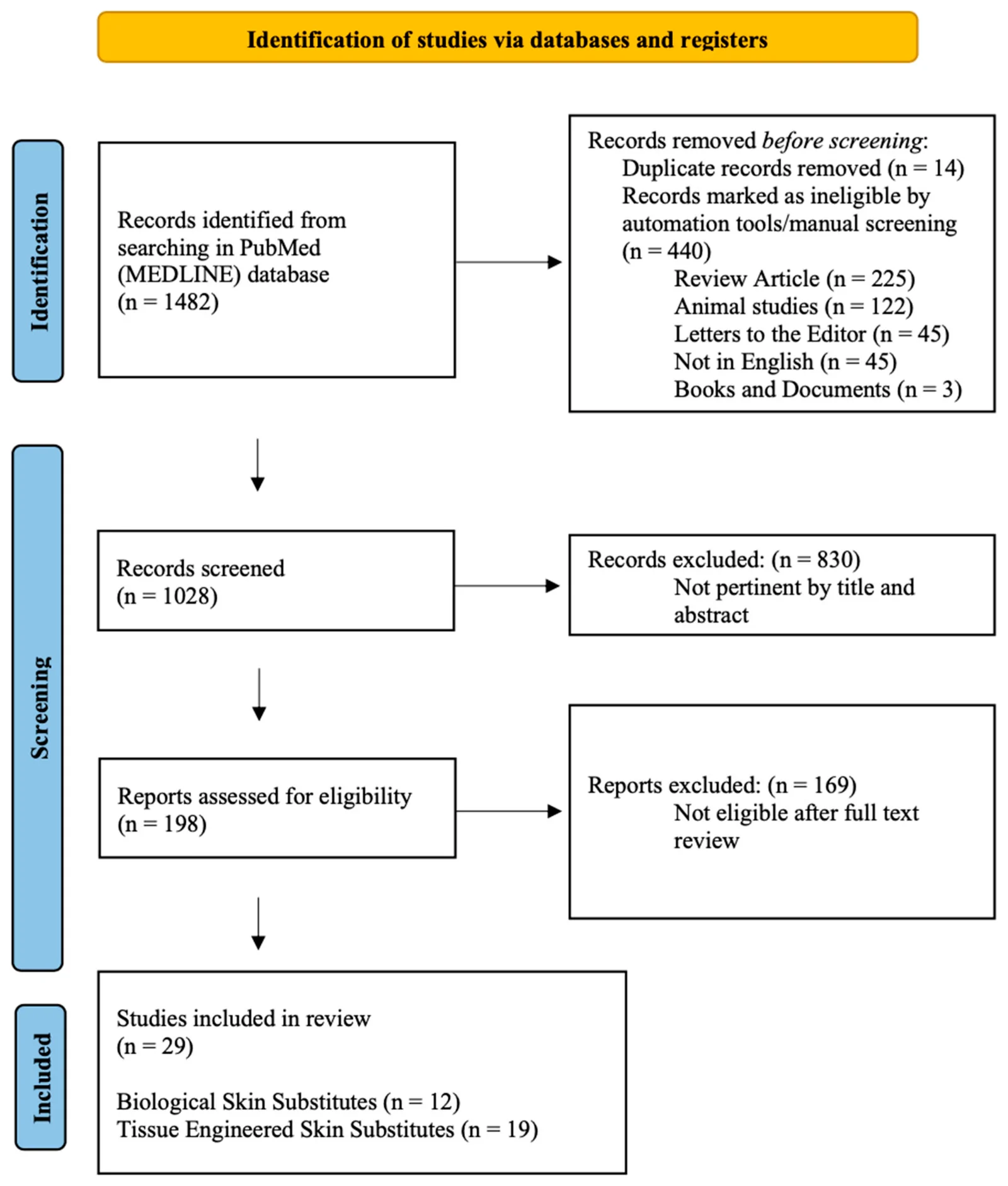
PRISMA flowchart.

Based on the established keywords, the primary research yielded a total of 1482 articles. These were compared to the selection criteria. By using PubMed’s automatic search tools and manual screening, 225 reviews and meta-analyses, 122 animal studies, 45 letters to the editor, 45 articles not written in English, and three books/documents were excluded. Fourteen duplicates were also excluded. The remaining articles were assessed for relevance based on their titles and abstracts; as a result, 198 potentially eligible original articles were selected and fully reviewed. Of these, 169 articles that were not relevant to the aim of this study were excluded. Finally, 29 articles met the selection criteria and were included in this review (Figure 1). These were classified into studies reporting on the role of BSS for pediatric burns (n. 12, Table 1) and studies on the role of TESS for pediatric burns (n. 17, Table 2).

Overall, the present review includes data on 2676 patients who underwent surgical reconstruction with SS due to partial-thickness or full-thickness pediatric burns. Outcomes are resumed in Table 3.

### 3.1. Outcomes for Biological Skin Substitutes

The BSS group (Table 1) includes 12 studies and 2210 patients (82.6% of all patients). Among these, 49.5% were females and 50.5% were males, with an average age of 4 (1–17), and an average TBSA of 22.9% (range 3–50%). Time to heal was specified for 83.4% of patients. Of these, the average time was 15 days (ranging from 10 to 26 days). Among BSS, we also extracted data on reconstructions by allograft. This subgroup included 5 studies, 86 patients (3.2%), 36.0% females, 64,0% males, average age 3 (1–17), average TBSA of 22.6% (range 6.8–31%). Time to heal was specified for 65.1% of patients. Of these, the average time was 16 days (ranging from 14 to 19 days).

### 3.2. Outcomes for Tissue Engineered Skin Substitutes

The TESS group (Table 2) includes 17 studies and 466 (17.4%) patients: 41.3% females, 58.7% males, average age 6 (1–17), average TBSA of 36.2% (3–73%). Time to heal was available for 239 patients. Of these, the average healing time was 15 days, ranging from 5 to 33 days. Among TESS, we also detailed results for pediatric burns reconstruction with Integra^®^. This subgroup included 5 studies, 85 patients (3.2%), 39.7% females, 60.3% males, average age 12 (7–16), average TBSA up to 73%. For the Integra^®^ subgroup, the time to heal was only specified by a study on 11 extended pediatric burns (TBSA of 495 ± 72 cm^2^) [40]. Complete healing was reported within 29 days for all patients, no major complications were reported [40].

When comparing the two main categories of skin substitutes, Biological Skin Substitutes (BSS) demonstrated an average healing time of 15 ± 5 days, while Tissue Engineered Skin Substitutes (TESS) showed a similar range (15 ± 6 days). However, the mean TBSA treated was higher among TESS studies (36.2%) than BSS (22.9%), suggesting preferential use of TESS in more extensive burns. Complication rates were generally low (<10%), with infection being the most frequent event. Among TESS, acellular matrices such as Integra^®^ and NovoSorb^®^ were associated with shorter healing times, whereas cellular products (OrCel™, Apligraf^®^) showed favorable long-term integration but were used in smaller series. Overall, trends indicate comparable safety and efficacy profiles between BSS and TESS, with acellular substitutes being the most widely adopted in pediatric practice.

Considering the lack of homogeneity of the studies included in this review and the difficulty in sub-analysis of the reported surgical techniques, additional data were not extracted. However, each strategy was critically analyzed and possibly discussed in the review.

## 4. Discussion

SS can be considered whenever autografting is contraindicated or unlikely to succeed. An ideal SS should simulate normal skin and offer firm adhesion to the receiving site, prevent dehydration, dyselectrolytemia, and wound drying, reduce microbial contamination, infection, and pain [22,23]. It should also represent a non-toxic, durable, and flexible dressing, easily and readily available at low cost [21,52,53]. To date, no product fulfills all these characteristics, but different strategies can be considered depending on the patient’s needs.

**Figure 2:**
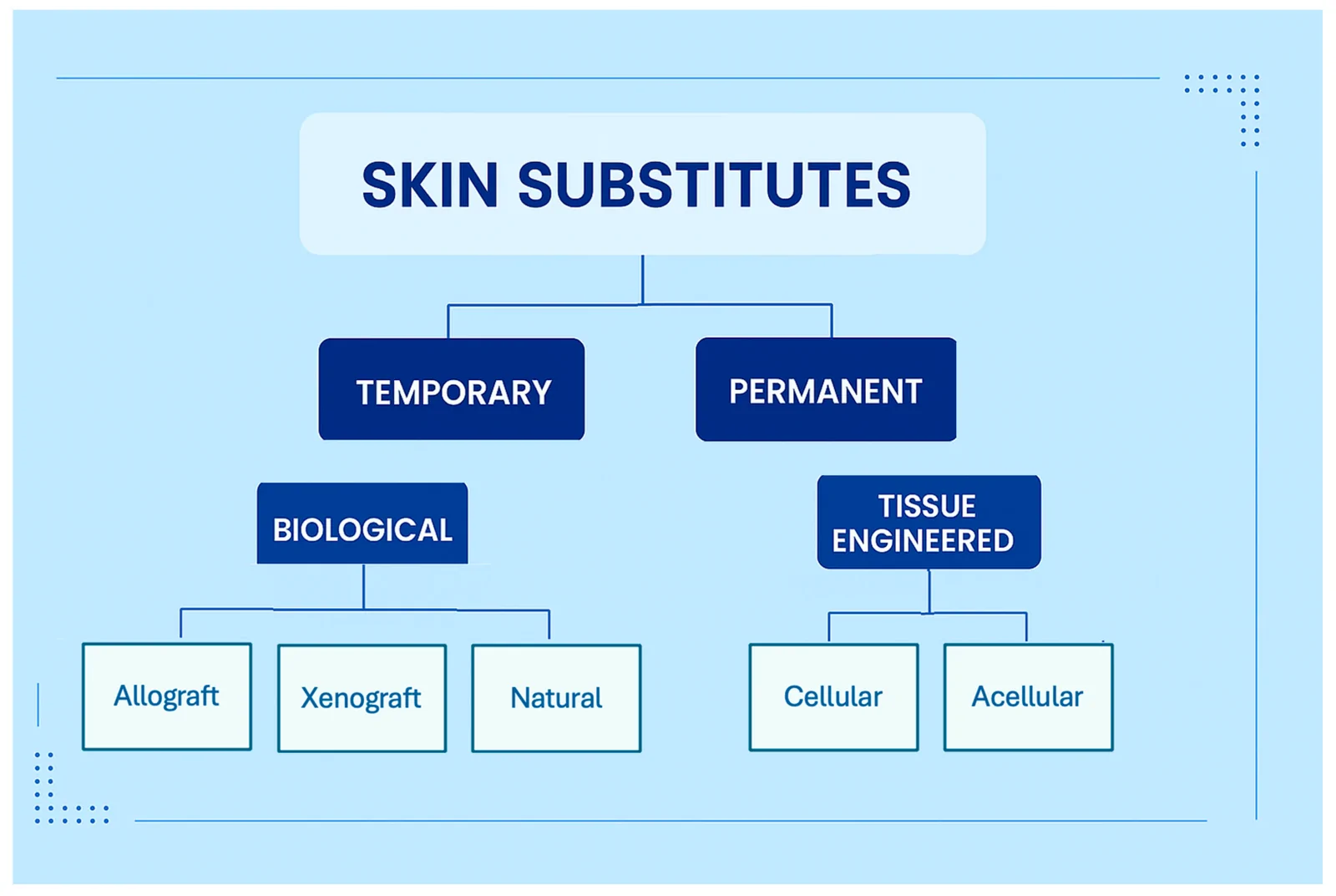
Skin substitutes classification.

Based on the literature review and the author’s personal experience, a schematic classification of SS is presented in Figure 2. First, temporary and permanent SS are available. Temporary SS represents an early coverage aimed at improving the wound bed before the secondary reconstruction. Permanent SS are fully integrated and represent a single-step reconstructive strategy. Based on the origin, SS can be classified into BSS and TESS. The former include allograft, xenograft, and natural SS (i.e., banana leaves [54], potato peel [55]). The latter can be further distinguished into acellular or cellular products.

### 4.1. Biological Skin Substitutes

#### 4.1.1. Allograft

First described by Bettman in 1938, a skin allograft is a temporary cover obtained from a human donor and preserved in skin banks [32,56]. Rejection is expected to begin within 1 to 2 weeks in immunocompetent recipients [57]. However, an allograft may last much longer in major burn patients, considering the burn-induced immunosuppression [58,59]. In some cases, the allograft is not rejected but progressively turned over and replaced by autologous tissue, offering a valuable scaffold for epidermal ingrowth [60,61,62].

In the current literature, we found evidence of 5 studies and over 86 pediatric burns effectively treated by allograft [27,29,31,32,34]. The procedure is clearly underreported, considering the widespread activity of skin banks [63]. The average healing time was 16 days (ranging from 14 to 19 days) for patients with an average TBSA of 22.6% (range 6.8–31%), and a few complications were reported (Table 1). Overall, allograft is a safe and effective procedure, and future research on a larger sample is expected.

#### 4.1.2. Amniotic Membranes

Among allografts, the amniotic membrane has been used as a BSS since 1910 [34]. It is obtained from the inner layer of the placentae of selected donors [34]. The amnion is preferred over the chorion, the outer layer of the placenta, due to lower antigenic properties [64].

Current indications include the treatment of partial thickness burns and damaged corneas [64,65]. Similarly to a skin allograft, it reduces dehydration, loss of electrolytes, pain, and infection. Specific advantages include a transparent biologic barrier that allows for direct visualization of the wound bed [66]. Among the disadvantages, it may require early replacement, after 2 or 3 days, due to progressive disintegration [66]. Moreover, it is quite difficult to obtain and store [64,65]. Notably, the necessity of donors’ written consent and comprehensive testing for transmissible diseases before childbirth is often responsible for the limited availability of amnion donors. Consistently, in addition to the fresh membrane, various preparations have been developed, including cryopreservation, glycerol preservation, freeze-drying, irradiation, and silver impregnation [60].

Among these, Dehydrated Human Amniotic/Chorionic Membrane (dHACM) is a non-immunogenic, anti-inflammatory, and antibacterial BSS that contains nonviable intact cells, chemokines, and cytokines that constitute a valuable scaffold for cellular migration, fibroblast recruitment, and proliferation [31,67]. It is widely adopted for the treatment of facial pediatric burns [67]. However, with respect to cadaveric skin allograft, there is still debate about the best strategy [68].

Technological advances in tissue engineering have also focused on the amniotic membrane. Hohlfeld et al. [45] in 2005 presented a bioengineered fetal skin construct of cultured fetal skin cells on native horse collagen. Specifically, 4 cm^2^ of skin allowed the preparation of several million three-dimensional skin constructs that were effectively employed for the treatment of eight pediatric burns [45]. Complete closure was obtained within 15 +/− 3 days without the need for delayed autografting. No retraction occurred, showing promising results.

#### 4.1.3. Xenograft

Xenograft consists of tissue transfer from one species to another [69,70,71]. The xenografts are prepared by cryopreservation, lyophilization, or chemical dehydration to minimize the immunogenic properties [70].

#### 4.1.4. Porcine Xenograft

Porcine xenografts have been largely adopted for the treatment of pediatric burns [71]. Various reports have documented similar or superior benefit compared to cadaveric allograft, considering the similar efficacy and low cost [18]. This temporary cover prevents dehydration and infection, while encouraging epidermal growth [69]. They are also bioactive SS. Indeed. The xeno-collagen matrix allows a temporary adherence to the wound. Reported disadvantages include lack of integration, rejection, and risk of infection [72].

#### 4.1.5. Tilapia Fish Skin

The Nile tilapia (Oreochromis niloticus) is the most cultivated fish in Brazil. Originally from East Africa, from the Nile River, it is currently distributed in most tropical and subtropical areas [28]. The tilapia skin resembles human skin; it presents a deep dermis, high ratios of thick, organized Type I collagen, and hosts a non-infectious microbiota [73]. It seems an innovative, easy-to-apply, highly available, pioneering product. Despite the promising evidence according to animal studies, it is currently poorly adopted for the treatment of pediatric burns [74].

### 4.2. Tissue Engineered Skin Substitutes

Tremendous advances have recently occurred in the field of TESS for wound healing. New products are now available in either synthetic or natural origin that are obtained from autologous, allogenic, xenogenic, or synthetic sources, including acellular materials [75]. Various preparations are available, such as cryopreservation, glycerol storage, lyophilization, and decellularization [23,52]. Biocompatibility remains a challenging issue, especially for SS that feature biological components, considering the risk of inflammation and rejection [23,52]. Schematically, TESS can be classified into acellular and cellular SS.

### 4.3. Acellular Tissue Engineered Skin Substitutes

#### 4.3.1. Integra^®^

Integra^®^ is an acellular TESS available since the 1980s; it is now widely adopted in plastic surgery for adults and children [76]. Common pediatric indications include surgical reconstruction of vascular malformations, congenital melanocytic nevi, aplasia cutis, myelomeningoceles, and burns [39,77].

Integra^®^ consists of a two-layer synthetic material. The superior layer emulates the epidermis. It includes an outer silicone membrane (polysiloxane polymer) that reduces bacterial colonization, infection, and wound drying, while allowing for vapor transmission [39,77]. The silicone layer is typically replaced with autograft or autologous epidermal culture after three weeks. The deeper layer resembles the skin dermis. It is composed of bovine cross-linked collagen and the glycosaminoglycan condroitin-6-sulfate [39,77]. These allow for endothelial cells and fibroblasts migration, ingrowth, and proliferation, acting as a bioactive scaffold. They also promote macrophages and lymphocytes infiltration, collagen deposition, neo angiogenesis and reinnervation [39,77].

In the field of pediatric burns, Integra^®^ has been demonstrated to effectively prevent burn scarring, secondary contracture, and donor site morbidity (Supplementary Figure S1) [77]. In addition, Integra^®^ is suitable for extensive postburn scar revisions [43]. We found 5 studies reporting data on 85 pediatric patients treated by Integra^®^ with average TBSA up to 73% [39,40,41,43,44]. Nessler et al. [40] reported complete healing within 29 days for all their patients presenting partial/full thickness extended burns (TBSA 495 ± 72 cm^2^). No major complications were reported [40]. Overall, this bilaminar structure justifies the possibility of reconstructing full-thickness (dermoepidermal) substance losses following burns, with excellent functional and esthetic outcomes [78,79].

#### 4.3.2. Biobrane^®^

Biobrane^®^, first developed in 1979, is a biosynthetic SS characterized by an outer ultrathin semipermeable epidermal analog (silicone film and knitted nylon mesh) and an inner dermal substitute made of Type 1 porcine collagen [80]. The latter promotes wound surface fibrin adherence [80]. Small pores have been designed to increase exudate drainage and permeability to topical antibiotics. Since Biobrane^®^ remains attached to the wound bed until epithelization occurs, daily debridement and related pain are avoided [80].

Current indications for Biobrane^®^ include temporary covering of superficial to mid-partial thickness burns, excised burns, donor sites, and protective covering over meshed autografts [80,81]. Furthermore, Biobrane^®^ allows for an excellent range of motion and tissue stretching. Consistently, it should be considered for burns on the skin overlying joints [80,81].

#### 4.3.3. AlloDerm^®^

AlloDerm^®^ is a processed acellular dermal matrix derived from human skin. By accurate tissue engineering, cellular elements are removed while retaining portions of the extracellular matrix [82]. Comparable to an allograft, it promotes fibroblast infiltration, neovascularization, and epithelialization. However, the inflammatory response is much limited and no rejection occurs, representing a valuable TESS for permanent dermis replacement [82]. In addition, AlloDerm^®^ can be grafted simultaneously with an overlying meshed autograft. Reported applications in children include oronasal fistula repair and partial- and full-thickness burns coverage [83,84].

#### 4.3.4. Matriderm^®^

Matriderm^®^ is a single-layer acellular TESS made of Type I, III, and V bovine dermal collagen and elastin hydrolysate organized in a highly porous structure. The collagen-elastine template acts as a bioactive scaffold promoting fibroblast proliferation, neoangiogenesis, and healing [85,86]. Then, it physiologically resorbs within 6 weeks [87]. Unlike most dermal SS, Matriderm^®^ is characterized by a non-cross-linked collagen [88]. Collagen cross-linking, as for Integra ^®^, has been related to increased rigidity, structural stability, and resistance to biodegradation of the dermal substitute [88]. However, cross-linking has also been related to increased fibroblast differentiation into myofibroblasts [88]. The latter have been demonstrated to play a role in post-burn wound contracture [89]. Consistently, Matriderm^®^ could be advantageous over cross-linked collagen TESS when faster resorption and less rigidity of the matrix are acceptable, but less wound contracture over time is desired.

#### 4.3.5. Kerecis^®^

The Atlantic cod (Gadus morhua) fish skin has been decellularized and effectively implicated as an acellular fish skin xenograft. Named Kerecis^®^, this TESS was recently approved by the United States Food and Drug Administration (FDA) in 2021 for the treatment of chronic wounds, burns, and other plastic surgery procedures, including pediatric indications (Supplementary Figure S2) [90].

Following meticulous fish skin processing, its structure and lipid composition are preserved. Specifically, Kerecis^®^ contains omega-3 polyunsaturated fatty acids. The latter have anti-inflammatory and antimicrobial properties that facilitate the transition from the post-burn inflammatory phase to the healing process [91]. In addition, Kerecis^®^ is characterized by a microporous structure that represents a valuable scaffold for tissue granulation, neo angiogenesis, and healing [91]. Such properties are beneficial in poorly vascularized body areas, such as the extremities.

In a study by Shahriari et al. [36], Kerecis^®^ was effectively incorporated into burned fingers, leading to optimal outcomes in terms of tissue elasticity, hand mobility, and cosmesis. Autografting and its related donor site morbidity were not required. Future research is warranted.

#### 4.3.6. NovoSorb^®^

Firstly introduced in 2015, NovoSorb^®^ Biodegradable temporizing Matrix (BTM) is a temporary TESS successfully tested in the adult population with various indications, including burns [92,93]. BTM is a biodegradable, entirely synthetic, 2 mm thick polyurethane foam, further sealed by a non-biodegradable polyurethane transparent membrane [94].

BTM requires a 2-stage approach. Firstly, the foam polymer is applied to the wound bed. Following matrix integration, cellular migration, neo angiogenesis, and collagen restoration, the transparent sealing layer is removed, leaving a well-vascularized neodermal layer [94]. The latter can be closed with an autograft. Reported indications include the treatment of partial and full thickness burns, traumatic injuries, necrotizing fasciitis, and free flaps donor sites reconstruction [92,94]. Recently, Storey et al. [37] reported BTM for the treatment of 19 pediatric burns. In their report, BTM successfully provided a temporary wound coverage, reducing the chance of local and systemic infections, as well as the need for the patient’s resuscitation and metabolic support. Overall, BTM has shown positive results for the treatment of pediatric wounds, and future research is expected.

#### 4.3.7. Cytal^®^ Burn Matrix

Cytal^®^ Burn Matrix is a medical device based on Urinary Bladder Matrix technology. It is an acellular TESS composed of intact epithelial basement membrane and collagen fibers [95]. Specifically, it is a porcine-derived urothelium, lyophilized and dehydrated. As a graft, Cytal^®^ promotes tissue remodeling and healing. It has been proposed for the treatment of second-degree burns in adults as well as vascular disease and fistula [96,97]. In the field of pediatric burns, its role is still to be defined (Supplementary Figure S3).

### 4.4. Cellular Tissue Engineered Skin Substitutes

#### 4.4.1. Autologous/Allogeneic Cultured Skin

Autologous/allogeneic cultured skin is based on collecting small samples of human skin and expanding them under artificial conditions, leading to a cultured TESS that retains the properties of the original skin [98]. Skin samples include both cellular components (mainly keratinocytes and fibroblasts, but also melanocytes) and an extracellular matrix. Potentially, from a simple biopsy, modern skin culture technologies allow for the replacement of the entire body surface within three to four weeks [98].

Current applications include the treatment of severe burns and skin pigmentation disorders (considering the presence of melanocytes), such as burn scars, ulcers, and vitiligo [98,99]. However, cultured keratinocytes and fibroblasts are currently poorly adopted due to limitations in terms of financial costs, access to a specialized biotechnology laboratory, and limited evidence [98].

#### 4.4.2. OrCel^TM®^

OrCel^TM®^ is a cellular biologic dressing. This bilayer matrix is composed of Type 1 bovine collagen sponge supporting live human allogeneic skin cells [48]. It includes human cultured skin epidermal keratinocytes and dermal fibroblasts obtained from human neonatal foreskin tissue [48]. The former are cultured on the coated non-porous side of the collagen matrix, while fibroblasts are cultured on the coated side. Both keratinocytes and fibroblasts are capable of proliferating and producing several growth factors and cytokines, leading to a favorable environment for tissue regeneration and healing [48].

Previous reports have documented the use of OrCel^TM®^ in the field of donor graft sites healing and hand contractures release in patients with recessive dystrophic epidermolysis bullosa [100]. OrCel^TM®^ has recently been involved in the treatment of pediatric burns with promising outcomes, and future research is expected [48].

#### 4.4.3. TransCyte^®^

TransCyte^®^ is a cellular TESS characterized by a semi-permeable silicone membrane (epidermal substitute) coupled with a porcine collagen-coated nylon mesh (dermal substitute) [47]. The former is a temporary epidermal layer that prevents dehydration, dyselectrolytes, and infection, easily removed once tissue healing has occurred. The latter includes a cellular component represented by cultured newborn human fibroblasts [47]. These proliferate and produce cytokines and growth factors [101]. TransCyte^®^ is also characterized by considerable pliability. Such physical property could be advantageous in cases of facial burns recontouring, following adequate non-viable tissue excision [101].

#### 4.4.4. Dermagraft^®^

Dermagraft^®^ is a sterile, cryopreserved, bioabsorbable TESS that includes an absorbable polymer scaffold (polyglycolic acid or polyglactin-910) coupled with allogeneic neonatal fibroblasts [102]. This latter produces dermal collagens (type I, III, and VII), proliferates and releases various proteins and factors, including glycosaminoglycans, fibronectin, and growth factors [102]. Dermagraft^®^ current indications include the treatment of venous and pressure ulcers, including diabetic foot ulcers [103]. In addition, Dermagraft^®^ can be considered as a temporary or permanent SS for pediatric burns [104].

#### 4.4.5. Apligraf^®^

Apligraf^®^ is a cellular bilayered TESS characterized by histological and handling characteristics such as human skin [105]. The epidermal layer presents human living keratinocytes organized into progressively differentiated layers, including a stratum corneum. The dermal substitute is based on Type I bovine collagen hosted by allogenic neonatal foreskin derived fibroblasts [105].

Animal studies have failed to identify any immune rejection across histocompatibility barriers or degradation phenomenon for up to 1 year [106]. The product has been approved by the FDA as a cellular TESS for the treatment of venous stasis ulcers [106]. In the field of pediatric burns, Apligraf^®^ can be considered as a temporary covering over meshed autograft following burn wound excision [107].

### 4.5. Comparative Interpretation and Evidence Ranking

The synthesis of available evidence suggests that BSS and TESS provide comparable short-term healing outcomes, although their clinical indications differ. BSS, including allografts and xenografts, are predominantly used for intermediate burns or as temporary coverage, while TESS are preferred for extensive or full-thickness injuries. Within TESS, acellular matrices (e.g., Integra^®^, NovoSorb^®^, Kerecis^®^) offer faster epithelialization and lower complication rates, whereas cellular substitutes (e.g., OrCel^TM^, Apligraf^®^) may promote better dermal remodeling at a higher cost and complexity. These comparative patterns, drawn from standardized outcome reporting, reinforce the complementary roles of BSS and TESS in pediatric burn reconstruction (Table 4).

Based on the included studies, the substitutes with the strongest pediatric clinical evidence are as follows: (1) Integra^®^ (acellular dermal regeneration template)—moderate evidence from multiple cohort studies and small RCTs supporting use for full-thickness defects and scar revision; (2) porcine xenografts/Biobrane^®^—moderate evidence supporting temporary coverage of partial-thickness burns and donor sites; (3) fish skin matrices (e.g., Kerecis^®^, tilapia skin)—growing evidence from case series and small trials for partial-thickness burns with advantages in pain and cost; (4) dehydrated human amnion/chorion membrane (dHACM)—several case series supporting facial and partial-thickness burns.

Cellular products (Apligraf^®^, OrCel^TM^, TransCyte^®^) have more limited pediatric series but show promise for donor-site management and as adjuncts over meshed grafts; however, they are used in smaller cohorts and are associated with higher complexity and cost.

Overall evidence strength across products remains heterogeneous (predominantly level III–IV), with only a minority of randomized data. Main limitations include cost, immunogenicity, storage, and ethics. For cost and access, many tissue-engineered and cellular products incur substantial cost and require specialized handling (cold chain, storage), limiting availability in resource-constrained settings. Integra^®^ and NovoSorb^®^ are relatively expensive but widely used in tertiary centers; fish skins and certain xenografts are lower cost and more available in some regions.

Concerning immunogenicity and infection risk, biological grafts (allograft, xenograft) can provoke immune responses and carry infection transmission concerns; however, decellularized and processed acellular matrices have substantially reduced immunogenicity. Reported infection rates in included studies were generally low but variable, and infection remains the most common reported complication.

For ethical and regulatory considerations, allograft use depends on donation systems and informed consent; xenografts may raise cultural/religious concerns; cellular products raise regulatory requirements for cell sourcing and manufacturing. These factors affect both research feasibility and clinical implementation in pediatric populations.

Finally, for specific pediatric considerations including growth, scarring, and long-term results), the pediatric skin is dynamic. Consistently, products must accommodate growth and avoid constrictive scarring that can impair function or limb development. Long-term follow-up is essential but lacking in most studies. Moreover, children are at higher risk for hypertrophic scarring and contractures; evidence suggests that dermal substitutes (e.g., Integra^®^) may reduce secondary contracture and improve scar quality, but definitive long-term comparative data are limited.

### 4.6. Study Limitations

The main limitation of this study is that it does not provide a complete treatment algorithm for pediatric burns. However, the paper offers a comprehensive assessment of SS, exploring the topic from various perspectives, based on the best available evidence.

Our results in terms of calculated healing time and reported complications should be interpreted as supporting evidence for the feasibility of the techniques rather than a parameter of efficacy. In fact, the data refer to an extremely heterogeneous population in terms of the size of the study sample, age of pediatric patients, and TBSA, preventing a true direct comparison. Moreover, in interpreting the results, it is important to consider that most studies included were observational, retrospective, or small case series, leading to an overall moderate-to-low level of evidence according to the JBI criteria. Despite this, the consistency of positive outcomes across independent reports supports the clinical feasibility and safety of BSS and TESS in pediatric burn care. Regarding the systematic review, it only included the PubMed (Medline) library, while other databases were not searched. This choice was deliberate to focus on clinically validated studies and the indexed medical literature, but it may have led to the omission of non-indexed reports. Moreover, the inclusion of heterogeneous study designs (case series, observational studies, RCTs) may introduce variability in outcomes and quality; this was mitigated through formal JBI risk-of-bias assessment. Furthermore, no statistical analysis was performed. Further investigations are warranted.

## 5. Conclusions

Surgical debridement and early excision of burned areas, along with skin replacement with autograft, currently represent the gold standard of treatment for partial and full-thickness pediatric burns. However, when skin autografting is contraindicated or unlikely to succeed due to inadequate conditions, SS should be considered.

Temporary and permanent SS of biological or tissue-engineered origin are all valuable options depending on the patient’s individual needs. Acellular and cellular TESS represent a new frontier in pediatric wound burn care. Numerous products are available in the market with positive data. This PRISMA-compliant systematic review highlights that, despite promising clinical results, the current evidence on BSS and TESS remains heterogeneous and largely descriptive.

Future high-quality multicenter studies and standardized reporting according to PRISMA and JBI frameworks are strongly encouraged to define evidence-based therapeutic algorithms for pediatric burn reconstruction. Further research is expected to investigate these substitutes with the aim of defining a therapeutic algorithm dedicated to the pediatric population.

## Figures and Tables

**Table 1:** Clinical evidence for the role of biological substitutes in pediatric burns (allograft, xenograft, and natural products).

Reference	N. Patients	Females	Males	Mean Age	TBSA (Mean)	Time to Heal (Days)	Complications	Study Field	Outcomes
Staubach et al. 2024 [26]	20	8	12	8	2.75%	26	Not available (NA)	Fish skin graft	Fish skin grafts can be considered for deep dermal pediatric burns.
Shen et al. 2021 [27]	22	9	13	5 (1–11)	31%, (10–86%)	15.5 (12–19)	NA	Fresh human skin allograft (scalp allografts from relatives)	Fresh scalp allografts from relatives are effective for major pediatric burns.
Lima Junior et al. 2020 [28]	30	12	18	5.7 ± 3.7	11.1% ± 4.9%	Silver Sulfadiazine: 10.5 ± 0.7 Tilapia fish skin: 10.1 ± 0.5	NA	Fish skin graft (Nile Tilapia fish skin)	Tilapia fish skin is an extra-low-cost alternative for pediatric partial-thickness burns.
Ahuja et al. 2020 [29]	30	14	16	3 (1–17)	NA	19.5 (15–35)	NA	dHACM	dHACM is a safe and feasible alternative to allograft for pediatric burns.
Costa et al. 2019 [30]	1	0	1	3	18%	10	None	Fish skin graft (Nile Tilapia fish skin)	Tilapia fish skin is a low-cost and widely available.
Puyana et al. 2019 [31]	30	7	23	3.7	6.8% (2–27%)	NA	None	dHACM	dHACM is a safe and feasible alternative to allograft for pediatric burns.
Gupta et al. 2019 [32]	1	1	0	5	30%	14	None	Fresh human skin allograft	Fresh human skin allograft is a cost-effective strategy.
Rode et al. 2017 [33]	35	NA	NA	4	49.7% (15–86%)	NA	1 graft failure (Acinetobacter baumanii infection)	Micrografting	Meek micrografting allows for high tissue expansion and durable wound cover.
Diegidio et al. 2017 [17]	1867	NA	NA	Autograft: 6 xenograft: 3	Autograft: 12.6% xenograft: 8.1%	NA	Infections autograf: 21 xeonograft: 4	Xenografting	Xenografting reduces the need for delayed reconstructions of partial-thickness burns.
Glat et al. 2017 [34]	3	0	3	2.3 (1–4)	NA	14 (7–21)	None	dHACM	dHACM is a safe and feasible alternative to allograft for pediatric burns.
Burkey et al. 2016 [18]	164	98	66	NA	7.0% (0.5–28%)	NA	4 (2.4%) infections	Porcine xenografting	Porcine xenografting can be considered for pediatric burns.
Menon et al. 2013 [35]	7	NA	NA	6	50% (range 30–70%)	NA	hypertrophic scarring graft loss (1–3%)	Micrograftin	Meek micrografting combined with cultured epithelial autograft (CEA) facilitates tissue expansion and wound closure.

**Table 2:** Clinical evidence for the role of tissue-engineered skin substitutes in pediatric burns.

Reference	N. Patients	Females	Males	Mean Age	TBSA (Mean)	Time to Heal (Days)	Complications	Study Field	Outcomes
Shahriari et al. 2024 [36]	1	1	0	1	3%	10	None	Fish skin graft (Kerecis^®^)	Kerecis^®^ was safe and effective.
Storey et al. [37]	19	NA	NA	7	2% (<1–46%)	NA	NA	NovoSorb^®^	NovoSorb^®^ is a safe and effective biodegradable, entirely synthetic, polyurethane foam.
Jackson et al. 2019 [38]	1	1	0	3	60%	33	None	Matriderm^®^	Matriderm^®^ combined with split skin grafts is effective for extensive facial burns.
Zajicek et al. 2017 [39]	28	NA	NA	NA	NA	NA	NA	Integra^®^	Integra^®^ improves scar quality in partial and full-thickness burns.
Yanaga et al. 2017 [20]	50	27	23	1–18	NA	9.3 (5–13)	Local infection: 4 Hypertrophic scar: 5	Cryopreserved cultured epithelial allograft	Cryopreserved cultured epithelial allograft is useful and effective.
Nessler et al. 2014 [40]	11	4	8	14 (12–16)	495 ± 72 cm^2^	29.1 ± 1.4 days	4 local infections	Integra^®^	Integra^®^ induces specific molecular patterns in pediatric burn healing.
Nessler et al. 2013 [41]	9	4	5	13	457.0 ± 65.1 cm^2^	NA	2 local infections 1 excessive granulation	Integra^®^	IL-4 and FGF levels may predict the development of complications following integra^®^ treatment.
Zajicek et al. 2011 [42]	86	NA	NA	NA	1–35%	NA	NA	Porcine xenograft (Xe-Derma^®^)	Acellular pig dermis Xe-Derma^®^ is effective for the treatment of scald burns.
Stiefel et al. 2009 [43]	17	11	6	13	NA	NA	2 seroma 1 hematoma	Integra^®^	Integra^®^ is safe and effective for burn scar revisions.
Branski et al. 2007 [44]	20	4	16	7	73%	NA	NA	Integra^®^	Integra^®^ allows for immediate burn wound cover and prevents cadaver-skin-related complications.
Hohfeld et al. 2005 [45]	8	5	3	4	NA	15·3 days (5·5)	Hypertrophic scars	Fetal skin TESS	Tissue-engineered fetal skin, repaired into three-dimensional constructs on horse-derived collagen, was a safe and effective permanent substitute.
Cassidy et al. 2005 [46]	72	34	38	NA	NA	Duoderm: 11.21 (+/−6.5) Biobrane: 12.24 (+/−5.1)	NA	TESS (Biobrane^®^)	Duoderm^®^ and Biobrane are equally effective for partial thickness pediatric burns. However, Duoderm^®^ is less expensive.
Kumar et al. 2004 [47]	33 (TransCyte, *n* = 20, Biobrane, *n* = 17; Silvazine, *n* = 21)	NA	NA	NA	NA	TransCyte—5; Biobrane—9.5; Silvazine—11.2	Failure to heal Silvazin: 5 (24%); Biobrane: 3 (17%); TransCyte: 1 (5%)	TransCyte^®^ vs. Biobrane^®^ vs. silvazine cream	TransCyte^®^ promotes faster epithelialization and easier dressings than Biobrane^®^ or silvazine cream.
Still et al. 2003 [48]	82	19	63	NA	10 to 80%	NA	NA	Bilayered living TESS (treatment of donor sites in burns)	OrCel^TM^^®^ contains proliferating keratinocytes and fibroblasts. It allows for a shorter healing time than Biobrane^®^.
Yanaga et al. 2001 [49]	43	23	19	5.1	30.7%	9.1 (6–12)	NA	Cryopreserved cultured epidermal allografts	Cryopreserved cultured epidermal allografts were effective in pediatric burns.
Waymack et al. 2000 [50]	Pediatric and adults	NA	NA	NA	NA	NA	NA	Bilayered living TESS (Apligraf)	Apligraf^®^ can be effectively applied over meshed autografts.
Barret et al. 2000 [51]	20	5	15	3	8.9% (+/−4.9%)	9.7 +/− 0.7	NA	TESS (Biobrane^®^)	Biobrane^®^ had superior outcomes compared to 1% silver sulfadiazine.

**Table 3:** Study population and skin substitutes subanalysis.

	Study Population		Skin Substitutes Subanalysis	
	Biological Skin Substitutes	Tissue Engineered Skin Substitutes	Allograft	Integra
N. studies	12	17	5	5
N. patients	2210 (82.6%)	466 (17.4%)	86 (3.2%)	85 (3.2%)
Females	49.5%	41.3%	36.0%	39.7%
Males	50.5%	58.7%	64.0%	60.3%
Mean Age/range	4 (1–17)	6 (1–17)	3 (1–17)	12 (7–16)
TBSA	22.9% (3–50%)	36.2% (3–73%)	22.6% (6.8–31%)	NA (up to 73%)
Time to heal (days)	15 (10–26)	15 (5–33)	16 (14–19)	NA (up to 29)

All averages were calculated as weighted means based on patient numbers per study. Only studies reporting complete outcome data (healing time, complications, and TBSA) were included in the comparative analysis.

**Table 4:** Comparative interpretation and evidence ranking for BSS and TESS.

Product/Class	Evidence Strength (Pediatric)	Main Pediatric Indications	Key Benefits (From Included Studies)	Key Limitations
Integra^®^ (acellular dermal template)	Moderate	Full-thickness reconstruction, scar revision, and extensive burns	Improved scar quality, reduces contracture; documented in several cohorts and small RCTs.	High cost; two-stage procedure; bovine collagen (xenogenic component).
Porcine xenografts/Biobrane^®^	Moderate	Temporary coverage of partial-thickness burns, donor sites	Readily available; reduces pain and dressing frequency; good short-term epithelialization.	Non-permanent; immunogenicity/rejection; cultural concerns.
Fish skin matrices (Kerecis^®^, tilapia skin)	Low–Moderate (growing)	Partial-thickness burns, hand burns	Low cost (tilapia); pain reduction; good epithelialization in small series.	Limited long-term data; variable processing/standards.
dHACM (dehydrated human amnion/chorion)	Low–Moderate	Facial/partial-thickness burns, adjuncts	Anti-inflammatory; easy handling; favorable cosmesis in case series.	Donor availability, variable preparations, and cost.
NovoSorb^®^ (synthetic BTM)	Low–Moderate	Complex wounds, temporary dermal replacement	Synthetic—no immunogenic cells; promising integration in pediatric case series.	Newer product—limited pediatric sample size; cost.
Allograft (human cadaveric/fresh scalp allograft)	Low–Moderate	Temporary coverage for extensive burns	Effective temporary cover; scaffold for healing.	Donor availability, disease transmission concerns, and variable longevity.
Cellular TESS (Apligraf^®^, OrCel^TM^, TransCyte^®^)	Low	Donor-site healing, adjunct to meshed autografts	Promote dermal remodeling; may shorten healing in select indications.	High cost, manufacturing complexity, and limited pediatric series.

Note: Evidence strength is derived from the number, design, and size of pediatric studies included in this review; “Moderate” does not imply high-quality randomized evidence but rather consistent clinical reports across multiple centers.

## Data Availability

The original contributions presented in the study are included in the article; further inquiries can be directed to the corresponding author.

## Supplementary Materials

The following supporting information can be downloaded at: https://www.mdpi.com/article/10.3390/jcm14227981/s1, Supplemental Digital Content 1: Prisma checklist; Figure S1: Two-year-old child. A: extended superficial second-degree flame burn. The burn extends to the deep dermis in the central and inferior-left side of the thorax. B: Integra^®^ graft. C: Follow up at 14 days; Integra^®^ has been completely integrated, reducing the area for skin autograft. D: follow up at 2 months; Figure S2: Two-year-old child. A: second-degree flame burn of the 4th and 5th rays of the left hand. B: Kerecis^®^ matrix graft. C, D: 30 days follow-up; complete healing without retracting sequelae; Figure S3: Two-year-old child presenting third-degree chest burn, TBSA 2%. A: surgical debridement and Cytal^®^. graft. B: Two-day follow-up. C: follow up at 5 days. Cytal^®^ is well integrated into local tissue; Table S1: Quality assessment of included studies according to the Joanna Briggs Institute (JBI) critical appraisal tools.

## Abbreviations

The following abbreviations are used in this manuscript:

BSS	Biological Skin Substitutes
BTM	Biodegradable Temporizing Matrix
CEA	Cultured Epithelial Autograft
dHACM	Dehydrated Human Amniotic/Chorionic Membrane
FDA	Food and Drug Administration
FGF	Fibroblast Growth Factor
HIV	Human Immunodeficiency Virus
HTLV	Human T-cell Lymphotropic Virus
IL-4	Interleukin 4
NA	Not Available
PICO	Population, Intervention, Comparator, Outcomes
PRISMA	Preferred Reporting Items for Systematic Reviews and Meta-Analyses
SS	Skin Substitutes
TBSA	Total Body Surface Area
TESS	Tissue Engineered Skin Substitutes
