# Supplementary material for: Pediatric Burns: Biological and Tissue Engineered Skin Substitutes—A Systematic Review
Source: J Clin Med. 2025 Nov 11;14(22):7981. doi: 10.3390/jcm14227981 (PMC12653667; doi:10.3390/jcm14227981)

Supplementary Table S1. Quality assessment of included studies according to the Joanna Briggs Institute (JBI) critical appraisal tools.

Reference (Author, Year)	Study Design	Sample Size (n)	JBI Checklist Applied	JBI Score (0–10)	Risk of Bias	Overall Quality	Main Limitations
Staubach et al., 2024	Case series	20	JBI Case Series	7/10	Moderate	Moderate	Small cohort, no control group
Shen et al., 2021	Prospective observational	22	JBI Cohort	8/10	Low–moderate	Good	Limited follow-up
Lima Junior et al., 2020	Pilot study	30	JBI Quasi-experimental	6/10	Moderate–high	Moderate	Lack of randomization
Ahuja et al., 2020	Retrospective cohort	30	JBI Cohort	7/10	Moderate	Moderate	No comparator arm
Costa et al., 2019	Case report	1	JBI Case Report	8/10	Low	High (single case)	Not generalizable
Puyana et al., 2019	Case series	30	JBI Case Series	7/10	Moderate	Moderate	Heterogeneous burn depth
Rode et al., 2017	Retrospective cohort	35	JBI Cohort	7/10	Moderate	Moderate	No randomization, variable TBSA
Diegidio et al., 2017	Retrospective cohort	1867	JBI Cohort	9/10	Low	High	Large sample, retrospective
Burkey et al., 2016	Case series	164	JBI Case Series	8/10	Low–moderate	Good	No standardized outcome measures
Menon et al., 2013	Prospective cohort	7	JBI Cohort	6/10	Moderate	Moderate	Small sample, short follow-up
Shahriari et al., 2024	Case report	1	JBI Case Report	8/10	Low	High (single case)	Limited external validity
Storey et al., 2023	Retrospective cohort	19	JBI Cohort	7/10	Moderate	Moderate	No control group
Jackson et al., 2019	Case report	1	JBI Case Report	7/10	Moderate	Moderate	Single case
Zajicek et al., 2017	Retrospective cohort	28	JBI Cohort	8/10	Low–moderate	Good	No randomization
Nessler et al., 2014	Case series	11	JBI Case Series	7/10	Moderate	Moderate	Limited reporting of confounders
Stiefel et al., 2009	Case series	17	JBI Case Series	7/10	Moderate	Moderate	Retrospective data collection
Branski et al., 2007	Randomized controlled trial	20	JBI RCT	9/10	Low	High	Small sample size
Hohlfeld et al., 2005	Pilot study	8	JBI Quasi-experimental	7/10	Moderate	Moderate	Limited to experimental model

Reference (Author, Year)	Study Design	Sample Size (n)	JBI Checklist Applied	JBI Score (0–10)	Risk of Bias	Overall Quality	Main Limitations
Cassidy et al., 2005	Randomized controlled trial	72	JBI RCT	9/10	Low	High	Short follow-up
Kumar et al., 2004	Randomized controlled trial	33	JBI RCT	8/10	Low	High	Limited randomization details

Supplementary Figure S1. Two-years old child. A: extended superficial second-degree flame burn. The burn extends to the deep dermis in the central and inferior-left side of the thorax. B: Integra® graft. C: Follow up at 14-days; Integra® has been completely integrated, reducing the area for skin autograft. D: follow up at 2 months.

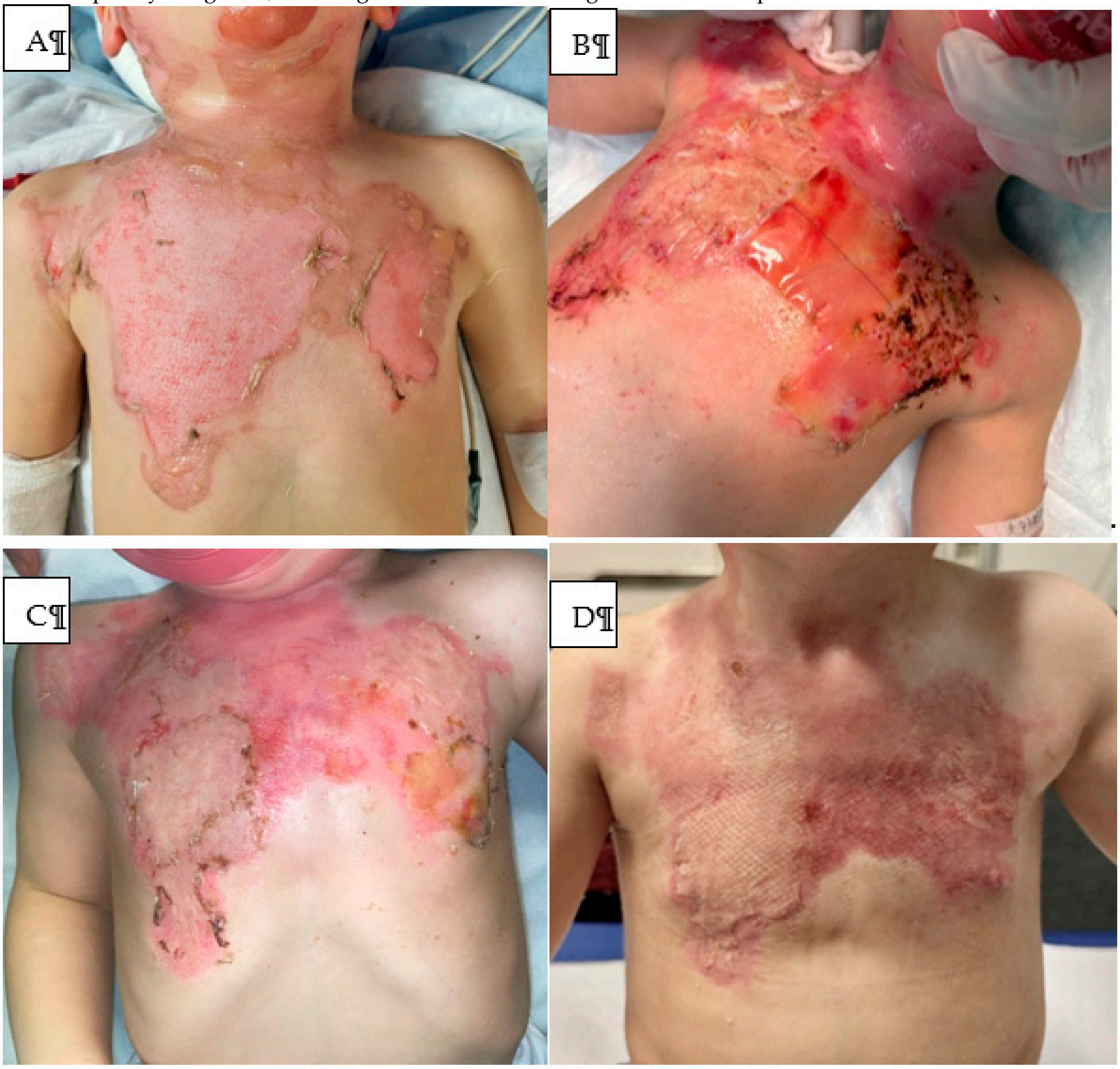

Supplementary Figure S2. Two-years old child. A: second degree flame burn of the 4th and 5th ray of the left hand. B: Kerecis® matrix graft. C, D: 30-days follow-up; complete healing without retracting sequelae.

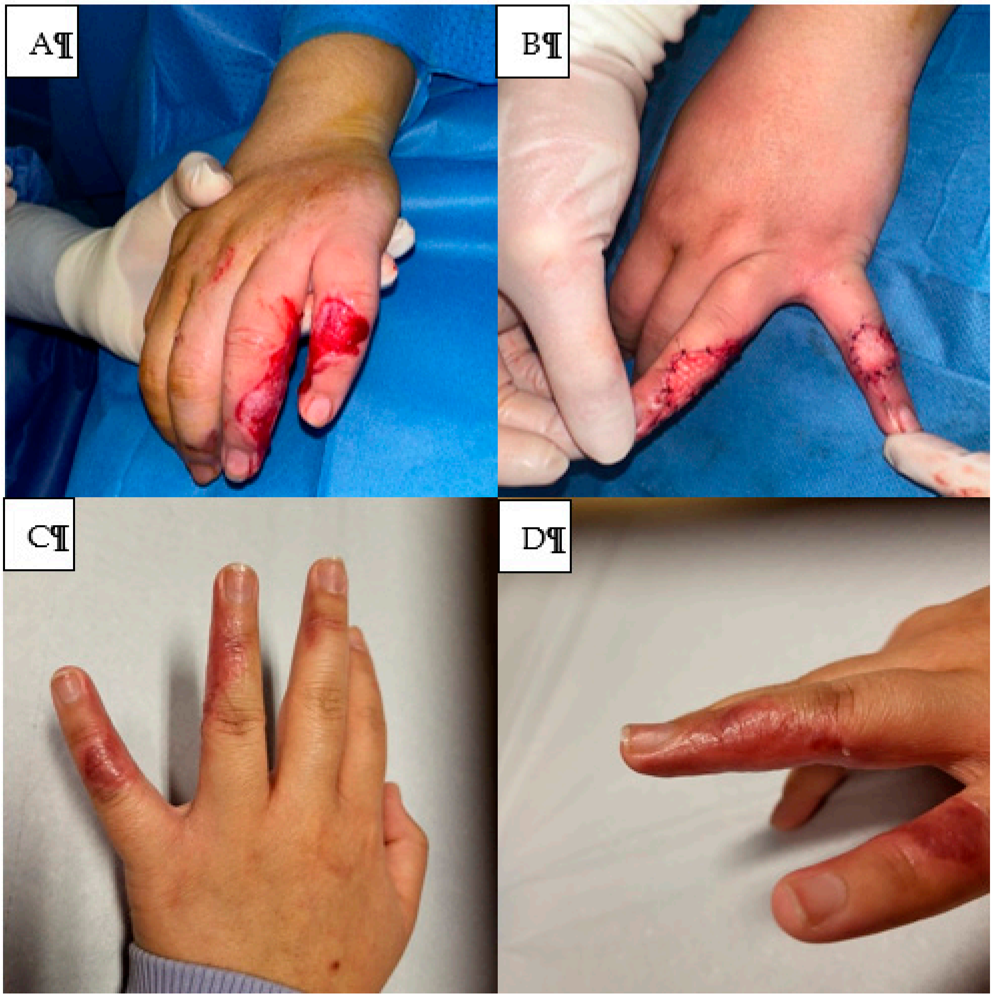

Supplementary Figure S3. Two-years old child presenting third-degree chest burn, TBSA 2%. A: surgical debridement and Cytal®. graft. B: two days follow-up. C: follow up at 5 days. Cytal® is well integrated into local tissue.

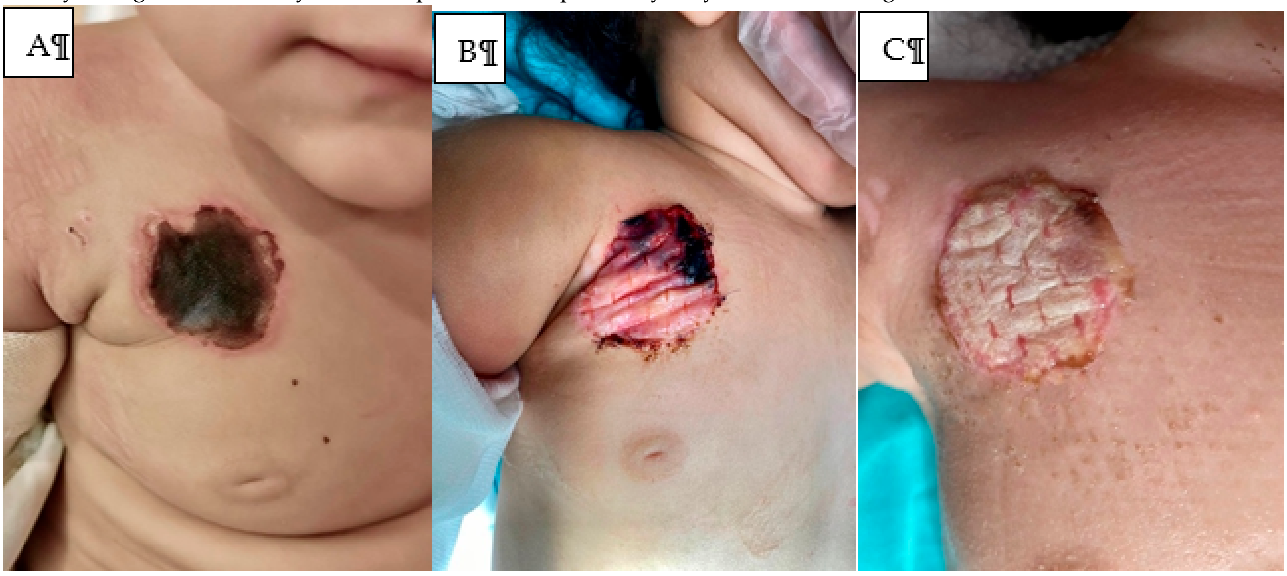

Supplement: Supplementary file 1 [file jcm-14-07981-s001.zip › jcm-3956679-supplementary-Table S1 and Figures.pdf]
